# Development of a Novel Aptamer-Antibody Sandwich Chemiluminescent Biosensor and Its Application in the Detection of Aflatoxin B_1_

**DOI:** 10.3390/bios15080538

**Published:** 2025-08-15

**Authors:** Zhike Zhao, Jianghao Feng, Caizhang Wu

**Affiliations:** 1 Key Laboratory of Grain Information Processing and Control, Henan University of Technology, Ministry of Education, Zhengzhou 450001, China; 2 Henan Key Laboratory of Grain Storage Information Intelligent Perception and Decision Making, Henan University of Technology, Zhengzhou 450001, China; 3 School of Electrical Engineering, Henan University of Technology, Zhengzhou 450001, China

**Keywords:** Aflatoxin B_1_, aptamer, chemiluminescence, indirect competition

## Abstract

In addressing the challenges posed by high costs, low accuracy, and cumbersome operations in mycotoxin detection, a novel aptamer-antibody sandwich chemiluminescent biosensor for detecting aflatoxin B_1_ (AFB_1_) was developed. The indirect competition between AFB_1_, aflatoxin B_1_-ovomucoid complete antigen (AFB_1_-OVA), and rabbit anti-ovomucoid (OVA) antibody results in the formation of a sandwich complex. This sandwich assay is linked to a horseradish peroxidase-labeled antibody, which catalyzes luminol chemiluminescence for the indirect detection of AFB_1_. The biosensor was designed to operate with high precision, low cost, and a low detection limit for AFB_1_, which is contingent upon experimental conditions such as pH, reagent concentration, temperature, and incubation time. The optimization of pH, aptamer concentration, competitive incubation time, competitive incubation temperature, and HRP-labeled antibody concentration was instrumental in achieving these objectives. Experimental findings demonstrated that the sensor’s detection limit was 0.067 ng/mL, exhibiting excellent linearity (R^2^ = 0.99679) within the concentration range of 0.25–10 ng/mL. The recovery rate of spiked samples ranged from 94.4% to 108.05%. This sensor boasts a low detection limit, straightforward operation, and minimal cost, thus offering a novel solution for developing cost-effective, high-precision mycotoxin detection methods.

## 1. Introduction

During the storage of agricultural products, fungi present in the products themselves and in the surrounding environment reproduce and metabolize, producing various mycotoxins that are highly toxic and carcinogenic, seriously affecting human health [1,2]. Fungal toxins that cause mold growth in various agricultural products include aflatoxin B_1_ (AFB_1_), aflatoxin B_2_ (AFB_2_), aflatoxin G_1_ (AFG_1_), aflatoxin G_2_ (AFG_2_), ochratoxin A (OTA), zearalenone (ZEA), deoxynivalenol (DON), T-2 toxin, etc. Mycotoxins have been identified as a major hazard to the safety of grain storage [3]. Aflatoxins are a diverse group of compounds that are widely present in crops such as peanuts, corn, soybeans, and wheat. Among them, AFB_1_ exhibits the highest toxicity and is not metabolizable [4,5]. AFB_1_ has been designated as a Group 1 carcinogen by the International Agency for Research on Cancer (IARC) [6,7]. According to statistics from the Food and Agriculture Organization of the United Nations (FAO), approximately 25% of agricultural products worldwide are contaminated with mycotoxins each year [8]. The presence of mycotoxins has emerged as a significant concern, posing a substantial threat to food safety. The World Health Organization (WHO) and the European Food Safety Authority (EFSA) have established regulations and guidelines that establish maximum limits for mycotoxin levels in food. The maximum permissible levels for aflatoxins in human and animal food are 0–20 nanograms per milliliter (ng/mL) and 0–50 ng/mL, respectively [9]. According to the prevailing Chinese standard, GB2761-2017, the maximum permissible content of aflatoxins in corn, corn flour, peanuts, and peanut oil is set at 20 µg/kg [10]. Consequently, the development of expeditious detection methodologies for mycotoxins, the reduction in detection expenses, and the enhancement of detection precision have become imperative to ensure food safety and to protect national economic interests [11].

The conventional methods for determining AFB_1_ in food primarily include high-performance liquid chromatography (HPLC) [12], liquid chromatography-mass spectrometry (LC-MS) [13], enzyme-linked immunosorbent assay (ELISA) [14], electrochemiluminescence (ECL) [15], fluorescence (FL) [16], fiber optic detection (FDDI) [17], and chemiluminescence (CL) [18]. Among the various types of biosensors, chemiluminescent biosensors are notable for their capacity to operate without an external light source. These biosensors provide several advantages, including low background interference, high sensitivity, rapid and straightforward analytical methods, cost-effectiveness, stable and reliable results, and minimal detection errors. Chemiluminescence immunoassay (CLIA) has been proven to be both convenient and reliable for detecting mycotoxins. The double-antibody sandwich method is a commonly used technique for identifying soluble antigens in chemiluminescent immunoassays. However, this approach is primarily suited for detecting high-molecular-weight targets rather than low-molecular-weight compounds (e.g., mycotoxins, antibiotics, and pesticides), as the two antibodies, which possess a significant steric hindrance, struggle to bind simultaneously to small-molecule targets. To illustrate this point, consider the example of AFB_1_, a small-molecule antigen. It is incapable of binding to bispecific antibodies due to the absence of two or more antibody binding sites. However, nucleic acid aptamers, which are capable of recognizing specific sequences of target molecules, can directly bind to low-molecular-weight compounds through their flexible structures, thereby achieving labeling or immobilization [19]. Aptamers are selected through a process known as “system-enriched ligand evolution technique” or SELEX for short. These peptides offer several advantages, including resistance to denaturation caused by temperature and pH changes, ease of storage, low cost, and the ability to be modified with different compounds. These properties make them suitable for a variety of applications, such as labeling and immobilization. These structures are formed through the process of nucleic acid molecular folding, which enables their highly specific binding to biological targets [20,21]. Consequently, aptamers are also referred to as “artificial antibodies.” At present, aptamer sensors represent a highly salient topic of research and a mainstream technological platform for the detection of mycotoxins. Scholars in both domestic and international contexts have conducted a series of related studies on aptamer sensors. Xie [22] et al. reported a novel enzyme-linked aptamer assay for the determination of AFB_1_. AFB_1_ competes with an immobilized biotin-aptamer for binding and subsequently releases biotin-complementary deoxyribonucleic acid (DNA), thereby inducing gradual fading of the color of the detection system as the concentration of AFB_1_ increases. The linear range of the detection method is 1–80 ng/mL, and the detection limit is 0.36 ng/mL. While this method was successful in detecting AFB_1_, its sensitivity was not optimal. Yang [23] et al. reported a fiber optic chemiluminescent aptamer sensor (FOCA) for the rapid and sensitive field detection of 17β-estradiol. E2-ovalbumin conjugate (E2-OVA) was covalently immobilized on an optical fiber, and the affinity constant of the E2/aptamer complex was determined using an optical fiber chemiluminescence aptamer sensor (FOCA). An indirect competitive assay for E2 detection was developed, achieving a detection limit of 0.18 nM. While this method is capable of detecting AFB_1_ with a high degree of efficiency and sensitivity, it necessitates the expertise of trained professionals and involves a series of complex steps. Tang [24] et al. developed a novel chemiluminescent resonance energy transfer (CRET) aptamer sensor for detecting OTA in corn. Based on gold nanoparticle (AuNP)-mediated CRET and Nb.BbvCI-powered DNA walker signal amplification. Signal amplification of the DNA walker driven by CRET and Nb.BbvCI was mediated by gold nanoparticles (AuNP). In AuNPs/Capture-DNA/HRP probes, HRP approaches the AuNP surface, causing CRET. AuNPs quench the chemiluminescence produced by HRP-catalyzed luminol. The detection limit of the CRET aptamer sensor is 2.75 nM, with a linear range of 5 to 40 nM. Although this method detects OTA, its sensitivity is not ideal, and it is not inexpensive. Zhang [25] et al. combined the properties of Co_3_O_4_ nanoparticles (Co_3_O_4_NPs) and highly specific DNA aptamers to construct a chemiluminescent aptamer sensor for the rapid quantitative detection of kanamycin (KAN). DNA aptamers/Co_3_O_4_NPs regulate chemiluminescence signals through the chemiluminescence properties of H_2_O_2_ oxidizing luminol. The concentration of KAN has a good linear relationship with the chemiluminescence signal intensity in the range of 0.5 to 8.0 μM, with a detection limit of 0.26 μM. This chemiluminescence sensor has the advantages of low cost, good specificity, and high sensitivity. Fytory [26] et al. proposed biosensors based on core-shell nanostructures formed by conducting polypyrrole (Ppy) and zirconium nanoscale metal-organic frameworks (Zr-NMOF). The aptamer sensor was used to detect ultra-trace OTA in food by altering the electrical properties of nanostructures, with a detection limit of 0.1 ng/L, a high dynamic linear range, and a high recovery rate compared with standard methods. Sun [27] and others developed a centrifugal microfluidic chip to simultaneously OTA, DON, and AFB_1_. The sensor is modified with MXene@AuNPs modified electrodes to obtain an excellent high-sensitivity detection performance. The detection limits of OTA, DON, and AFB_1_ were 7.1 ng/L, 1.24 ug/L, and 11.8 ng/L, respectively, and the average recoveries of the method ranged from 94.0% to 106.1%. Yin [28] et al. proposed a chemiluminescence/colorimetric dual-signal microfluidic chip combined with streptavidin-biotin-alkaline phosphatase signal amplification for the indirectly competitive and highly sensitive detection of DON, OTA, and AFB_1_. The sensitive detection range of the sensor for DON was 4–128 ng/mL, OTA was 2–64 ng/mL, and AFB_1_ was 0.2–6.4 ng/mL, and the detection limits were 2.636 ng/mL, 1.492 ng/mL, and 0.131 ng/mL, with the recoveries ranging from 91.93% to 109.31%, respectively.

Therefore, this study combined the advantages and disadvantages of the above sensors to develop an aptamer-antibody sandwich chemiluminescent biosensor for the high-precision detection of AFB_1_. In this study, amino aptamers were conjugated to carboxyl magnetic nanoparticles to achieve coating of the amino aptamers. AFB_1_ competes with AFB_1_-OVA for binding to the amino aptamers conjugated to the carboxyl magnetic nanoparticles. Rabbit anti-OVA polyclonal antibodies were used to bind AFB_1_-OVA, forming an aptamer-antibody sandwich. Chemiluminescence was achieved by using horseradish peroxidase-labeled secondary antibodies to catalyze luminol and hydrogen peroxide. This study employs the principle of an indirect competitive sandwich immunoassay to achieve the indirect detection of AFB_1_ using the sandwich method. It utilizes specific aptamers and aptamer-antibody sandwich bispecific recognition, combined with a signal amplification strategy (horseradish peroxidase-labeled secondary antibody), to achieve the highly specific and precise detection of AFB_1_.

## 2. Materials and Methods

### 2.1. Reagents and Materials

AFB_1_ sample dilution solution, carboxyl magnetic beads (particle size 1.5 μm), luminol, H_2_O_2_, and p-iodophenol were purchased from Antu Bio (Zhengzhou, China). AFB_1_-OVA was purchased from Antigen Biotech Co., Ltd. (Shenzhen, China). Deionized water was purchased from Suzhou Weili Environmental Protection Technology Co., Ltd. (Suzhou, China).

N-Hydroxy succinimide (NHS), 1-(3-Dimethylaminopropyl)-3-ethylcarbodiimide (EDC), Anhydrous methanol, Bovine serum albumin (BSA), Tween 20, MES buffer (pH 5.0), PBS phosphate-buffered saline (1X, pH 7.4), TE buffer (1X, low EDTA, and pH 8.0), Rabbit anti-chicken ovalbumin (anti-OVAL), and HRP-labeled goat anti-rabbit IgG were purchased from Shanghai Sangon Biotech Co., Ltd. (Shanghai, China). The sequence of the AFB_1_ amino aptamer (NH_2_-AFB_1_-Aptamer) was synthesized by Shanghai Sangon Biotech Co., Ltd. (Shanghai, China) and purified using high-performance liquid chromatography (HPLC). The aptamer sequence (5′ to 3′) is as follows: 5′-NH2 C6-GT TGG GCA CGT GTT GTC TCT CTG TGT CTC GTG CCC TTC GCT AGG CCC ACA-3′; the 5′ end is modified with an amino group.

### 2.2. Measurement and Instruments

A Mettler Toledo electronic analytical balance was purchased from Mettler Toledo Instruments Co., Ltd. (Shanghai, China). The HH-2 digital constant temperature water bath was purchased from Bangxi Instrument Technology Co., Ltd. (Shanghai, China). The pipette was purchased from Shanghai Hongzun Medical Equipment Co., Ltd. (Shanghai, China). The 96-well enzyme-linked immunosorbent assay plate was purchased from AnTu Bioengineering Co., Ltd. (Zhengzhou, China). The ultrasonic cleaning machine was purchased from Shenzhen Jiemeng Cleaning Equipment Co., Ltd. (Shenzhen, China). The high-speed centrifuge was purchased from Shanghai Lichengbangxi Instrument Technology Co., Ltd. (Shanghai, China). The ZW-A micro-oscillator was purchased from Changzhou Runhua Electric Co., Ltd. (Changzhou, China). The H10682-210 Photon Counting Probe Module was purchased from Hamamatsu Photonics Trading Co., Ltd. (Beijing, China). The fiber optic mycotoxin detection system was developed independently.

### 2.3. AFB_1_ Sample Solution Pretreatment

A methanol-water solution (10:90, *v*/*v*) served as the diluent. AFB_1_ solution with a concentration of 1 μg/mL in a methanol-water solution (10:90, *v*/*v*) was diluted to obtain different AFB_1_ solutions with concentrations of 0.25, 0.5, 1, 5, and 10 ng/mL. The stock methanol-water solution (10:90, *v*/*v*) without AFB_1_ served as the 0 ng/mL solution. The resulting AFB_1_ sample solutions of different concentrations were stored at 4 °C for later use.

### 2.4. Preparation of Carboxyl Magnetic Beads Coated with AFB_1_ Amino Aptamers

**Figure 1:**
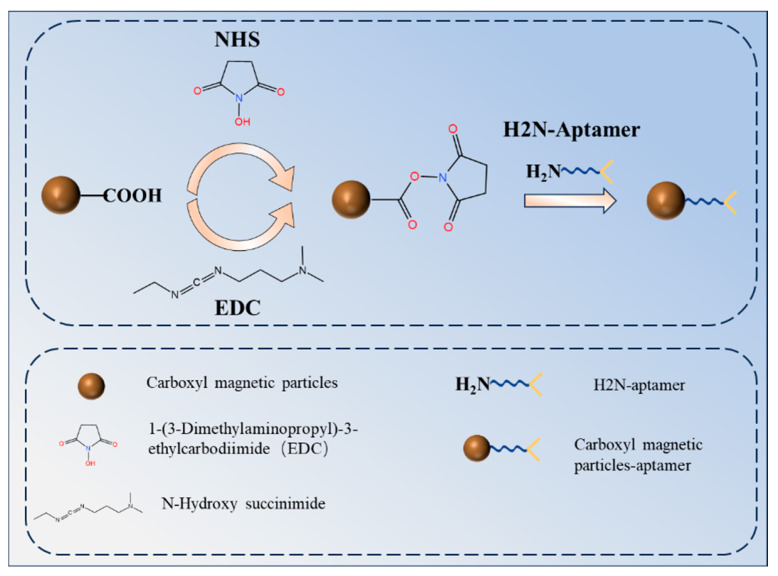
Schematic diagram of the process of coating carboxyl magnetic microparticles with amino aptamers.

A total of 20 μL of carboxyl magnetic bead solution with a concentration of 10 mg/mL was added to a centrifuge tube. Following vortex mixing for 15 s, the beads were positioned on a magnetic separation rack, allowing the supernatant to be removed via magnetic separation. The magnetic bead solution was subsequently washed three times with 200 μL of MES buffer (pH 5.0) while remaining on the magnetic separation rack. After 3 min of magnetic separation, the supernatant was removed. A total of 10 mg each of EDC and NHS powders were dissolved in 1 mL of MES solutions, respectively, to prepare EDC and NHS solutions with a concentration of 10 mg/mL each. A total of 100 μL of EDC solution (10 mg/mL) and NHS solution (10 mg/mL) were added to the centrifuge tube containing the carboxylated magnetic bead solution. A ZW-A micro-oscillator was used to activate the solution by oscillating it at 25 °C under light-protected conditions for 30 min. The activated magnetic bead solution was washed three times with 200 μL of MES buffer (pH 5.0) to remove any remaining EDC and NHS solution. A total of 100 μL of AFB_1_ aptamer solution (1 nM) was added to the activated magnetic bead solution in addition to 200 μL of BSA blocking solution. The beads were allowed to coat at 4 °C for 14–16 h. A schematic diagram of the process of coating carboxyl magnetic microparticles with amino aptamers is shown in Figure 1.

Figure 1 depicts the process of coupling amino aptamers by carboxylated magnetic beads. The addition of EDC and NHS activates the carboxyl group to form a stable NHS ester, which forms a stable amide bond with the amino aptamer, thus completing the coupling of the carboxyl beads to the aminodentate ligand.

### 2.5. Chemiluminescent Aptamer Indirect Competitive Assay AFB_1_

A total of 200 μL of PBS solution containing 0.05% Tween-20 (PBST) was used to wash the coated solution three times. A total of 200 μL of BSA blocking solution was added to a centrifuge tube and incubated at 37 °C in a constant temperature water bath for 1 h to block unbound activated carboxyl groups. A total of 1 μg/mL AFB_1_-OVA reagent was diluted with PBS solution and stored for later use. A total of 100 μL of AFB_1_ diluent of varying concentrations (0, 0.25, 0.5, 1, 5, and 10 ng/mL) was added and another 100 μL of AFB_1_-OVA diluent at a concentration of 1 μg/mL to the centrifuge tube. The mixture was incubated at 30 °C for 30 min for competitive binding of the aptamer. The mixture was placed on a magnetic separation rack. After 3 min, unbound AFB_1_ and AFB_1_-OVA were removed by washing 5 times with PBST solution.

A total of 100 μL of rabbit anti-OVAL polyclonal antibody solution with a concentration of 1 μg/mL was placed in each centrifuge tube and incubated at 30 °C for 30 min. This mixture was placed on a magnetic separation rack for 3 min. The unbound rabbit anti-OVAL antibody was removed by washing 5 times with PBST solution.

A total of 100 μL of HRP-labeled goat anti-rabbit IgG solution with a concentration of 400 ng/mL was added to each centrifuge tube and incubated at 37 °C for 30 min. The mixture was placed on a magnetic separation rack for 3 min. The unbound HRP-labeled goat anti-rabbit solution was removed by washing 5 times with PBST solution.

A total of 200 μL of the luminescent substrate mixture solution (2 mM luminol, 0.5 mM p-iodophenol, and 2 mM H_2_O_2_) were added to each centrifuge tube. Optical fibers were used to detect the solution in the centrifuge tubes in a dark environment. The chemiluminescent intensity was measured using a self-made optical fiber detection system.

## 3. Results

### 3.1. Principle of Chemiluminescent Biosensor Detection of AFB_1_

**Figure 2:**
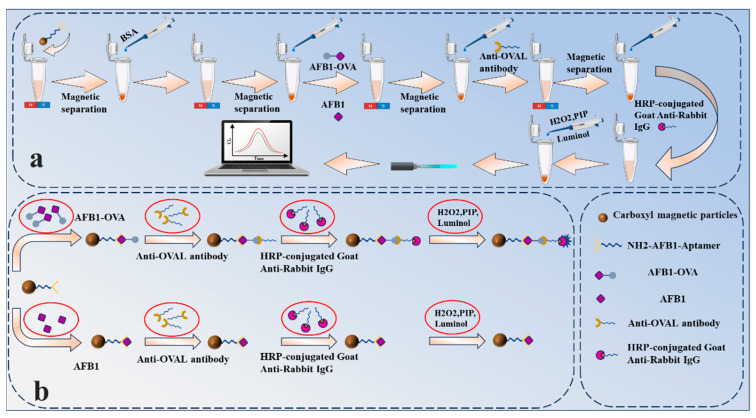
(**a**) Schematic diagram of the AFB_1_ chemiluminescence detection process; (**b**) schematic diagram of the mechanism of competitive binding of AFB_1_ and AFB_1_-OVA to the aptamer.

Figure 2 describes the chemiluminescent aptamer detection process for AFB_1_ in this experiment. Carboxyl magnetic beads were selected as the solid phase carrier, and EDC and NHS solutions were used to activate the carboxyl groups on the surface of the magnetic beads. After the carboxyl groups on the surface of the magnetic beads are activated, the AFB_1_ amino aptamer solution is added. The AFB_1_ aptamer modified with amino groups at the 5′ end can bind to the carboxyl groups on the surface of the magnetic beads to form a magnetic bead-aptamer complex. After removing the unbound aptamer solution by magnetic separation, the AFB_1_ toxin and AFB_1_-OVA complete antigen are added to the solution, and AFB_1_ and AFB_1_-OVA compete for binding to the aptamer. After competitive binding is complete, magnetic bead-aptamer-AFB_1_ complexes and magnetic bead-aptamer-AFB_1_-OVA complexes are formed in the solution. After removing unbound AFB_1_ from the AFB_1_-OVA mixture solution by magnetic separation, the rabbit anti-OVAL polyclonal antibody solution is added. Rabbit anti-OVAL polyclonal antibodies cannot recognize AFB_1_ but can specifically recognize AFB_1_-OVA and bind to AFB_1_-OVA to form a magnetic bead-aptamer-AFB_1_-OVA-rabbit anti-OVAL antibody complex. After removing the unbound rabbit anti-OVAL antibody solution by magnetic separation, HRP-labeled goat anti-rabbit IgG solution is added. HRP-labeled goat anti-rabbit IgG can specifically bind to the rabbit anti-OVAL antibody, forming a magnetic bead-aptamer-AFB_1_-OVA-rabbit anti-OVAL polyclonal antibody-HRP-labeled goat anti-rabbit IgG complex. Finally, the immune complex is placed in the dark, and a chemiluminescent substrate mixture (luminol, p-iodophenol, and H_2_O_2_) is added, and the solution will emit a blue glow. The chemiluminescence detection system developed in-house transmits chemiluminescence signals via optical fiber to a photon counter, which converts the chemiluminescence signals and outputs them to a microcontroller. The microcontroller counts the signals and outputs them to a computer.

This experiment uses an indirect competition method to indirectly detect the AFB_1_ concentration by detecting the formed magnetic bead-aptamer-AFB_1_-OVA-rabbit anti-OVAL-antibody-HRP-labeled goat anti-rabbit IgG complex. When AFB_1_ competes with AFB_1_-OVA for binding to the aptamer, the higher the concentration of AFB_1_, the more AFB_1_-aptamer complexes are formed, and the fewer aptamer-AFB_1_-OVA complexes are formed. The less the rabbit anti-OVA antibody specifically binds to AFB_1_-OVA results in fewer magnetic bead-aptamer-AFB_1_-OVA-rabbit anti-OVAL-antibody-HRP-labeled goat anti-rabbit IgG complexes formed and consequently lowers luminescence intensity. Conversely, the lower the AFB_1_ concentration, the fewer AFB_1_-aptamer complexes are formed, resulting in more aptamer-AFB_1_-OVA complexes. This leads to more rabbit anti-OVAL antibodies specifically binding to AFB_1_-OVA, ultimately forming more magnetic bead-aptamer-AFB_1_-OVA-rabbit anti-OVAL antibody-HRP-labeled goat anti-rabbit IgG complexes, thereby increasing the luminescence intensity.

### 3.2. Feasibility of AFB_1_ Detection Using Chemiluminescent Biosensors

**Figure 3:**
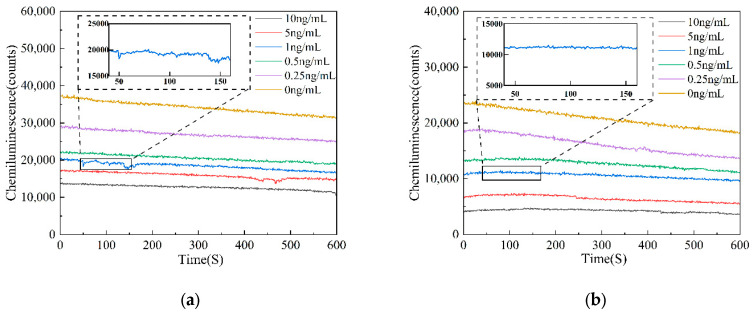
AFB_1_ detection curve diagram. (**a**) Microplate as solid-phase carrier detection curve diagram; (**b**) magnetic particles as solid-phase carrier detection curve diagram.

This study compared AFB_1_ reagents at concentrations of 0, 0.25, 0.5, 1, 5, and 10 ng/mL under two distinct solid-phase carrier conditions: microplates and magnetic microparticles. The objective was to verify the feasibility of the biosensor for AFB_1_ chemiluminescence detection. Different concentrations of AFB_1_ reagents were continuously tested for a duration of 10 min. The detection results indicate that the luminescence intensity observed with microplates as solid-phase carriers is relatively strong; however, the detection curve exhibits significant fluctuations, indicating poor stability. In contrast, when magnetic microparticles serve as solid-phase carriers, the detected luminescence intensity is comparatively weak, yet the detection curve demonstrates high stability and accuracy. The detection results are shown in Figure 3.

### 3.3. Optimization of Experimental Conditions

**Figure 4:**
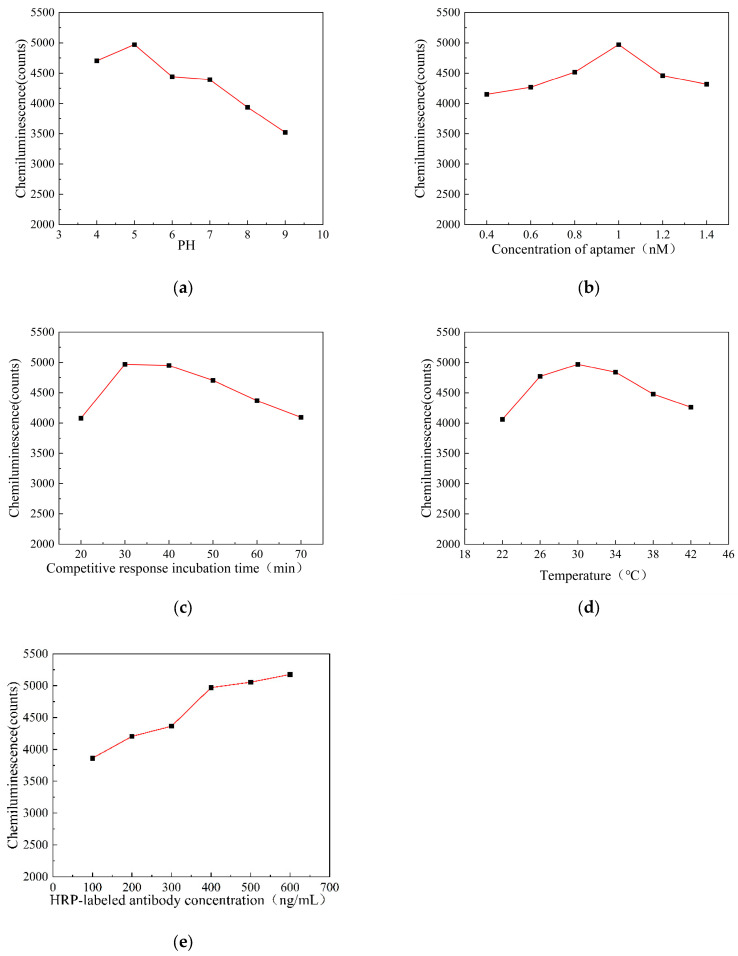
(**a**) pH affects the chemiluminescence intensity curve; (**b**) aptamer concentration affects the luminescence intensity curve; (**c**) competitive incubation time affects the luminescence intensity curve; (**d**) competition incubation temperature affects the luminescence intensity curve; and (**e**) HRP-labeled antibody concentration affects the luminescence intensity curve.

In order to improve the analytical performance of the chemiluminescence aptamer detection of AFB_1_ and obtain the best signal response, it is necessary to optimize the experimental conditions affecting the chemiluminescence intensity. The chemiluminescence intensity is affected by many factors, which mainly include the pH value when activating the carboxyl groups on the surface of the magnetic particles, the concentration of the aptamer, the incubation time of the competition reaction, the incubation temperature of the competition reaction, and the concentration of the HRP-labeled antibody. The experimental condition optimization curve is shown in Figure 4.

The activation efficiency of carboxyl groups on the surface of magnetic microparticles has a significant impact on the detection performance of sensors. This experiment investigated the effect of different pH values (4, 5, 6, 7, 8, and 9) on the luminescence intensity of AFB_1_ at a detection concentration of 10 ng/mL when the carboxyl groups on the surface of magnetic microparticles were activated. The changes in chemiluminescence intensity at different pH values are shown in Figure 4a. The chemiluminescence intensity reaches its peak at a pH of 5.0 and decreases after a pH of 7.0. This may be because EDC rapidly hydrolyzes and becomes ineffective in an alkaline environment, unable to effectively activate the carboxyl groups on the surface of magnetic particles, resulting in the low coupling efficiency of the aptamer and a decrease in chemiluminescence intensity. Therefore, a pH value of 5.0 was selected as the optimal pH value for the activation of carboxyl groups on the surface of magnetic microparticles.

The aptamer provides a binding site for the AFB_1_ antigen in the immune response, so the sensitivity of the sensor depends on the concentration of the aptamer fixed on the surface of the magnetic microparticles. This experiment investigated the effect of competitive binding at different aptamer concentrations (0.4, 0.6, 0.8, 1.0, 1.2, and 1.4 nM) on the luminescence intensity of the sensor detecting AFB_1_ at a concentration of 10 ng/mL. The changes in chemiluminescence intensity at different aptamer concentrations are shown in Figure 4b. The chemiluminescence intensity increases with the increasing aptamer concentration up to 1.0 nM. However, when the aptamer concentration exceeds 1.2 nM, the chemiluminescence intensity decreases. This may be because the concentration of aptamers immobilized on the magnetic beads has reached saturation. Therefore, 1.0 nM was selected as the optimal ligand concentration.

The duration of competitive binding of AFB_1_ and AFB_1_-OVA to the aptamer also has a significant impact on the sensitivity of the sensor. This experiment investigated the effect of the duration of competitive binding of the aptamer from 20 min to 70 min on the chemiluminescence intensity of AFB_1_ at a concentration of 10 ng/mL. The changes in chemiluminescence intensity under different competitive binding durations are shown in Figure 4c. The chemiluminescence intensity reaches its peak at 30 min of competition time and gradually decreases after 30 min. Therefore, 30 min was selected as the optimal duration for the competitive combination reaction.

The incubation temperature of AFB_1_ with the AFB_1_-OVA competition binding aptamer also has a strong effect on the sensitivity of the sensor. This experiment investigated the effect of different incubation temperatures (22, 26, 30, 34, 38, and 42 °C) on the detection of AFB_1_ chemiluminescence intensity at a concentration of 10 ng/mL when competing for the binding aptamer. The changes in chemiluminescence intensity at different incubation temperatures during the competition for binding aptamers are shown in Figure 4d. The chemiluminescence intensity peaks at an incubation temperature of 30 °C during competitive binding and decreases after 30 °C. Therefore, 30 °C was chosen as the optimal incubation temperature for the competitive binding reaction.

The sensitivity of the sensor is also affected by the concentration of HRP-labeled antibodies. This experiment investigated the effect of different HRP-labeled antibody concentrations (100, 200, 300, 400, 500, and 600 ng/mL) on the chemiluminescence intensity of AFB_1_ at a detection concentration of 10 ng/mL. The changes in chemiluminescence intensity at different HRP-labeled antibody concentrations are shown in Figure 4e. The chemiluminescence intensity increases with the increasing antibody concentration up to 400 ng/mL. When the concentration exceeds 400 ng/mL, the increase in chemiluminescence intensity slows down and gradually stabilizes. Considering both reagent consumption and luminescence intensity, 400 ng/mL was selected as the optimal concentration for HRP-labeled antibodies.

### 3.4. Performance Evaluation of Chemiluminescent Aptamer Sensors

**Figure 5:**
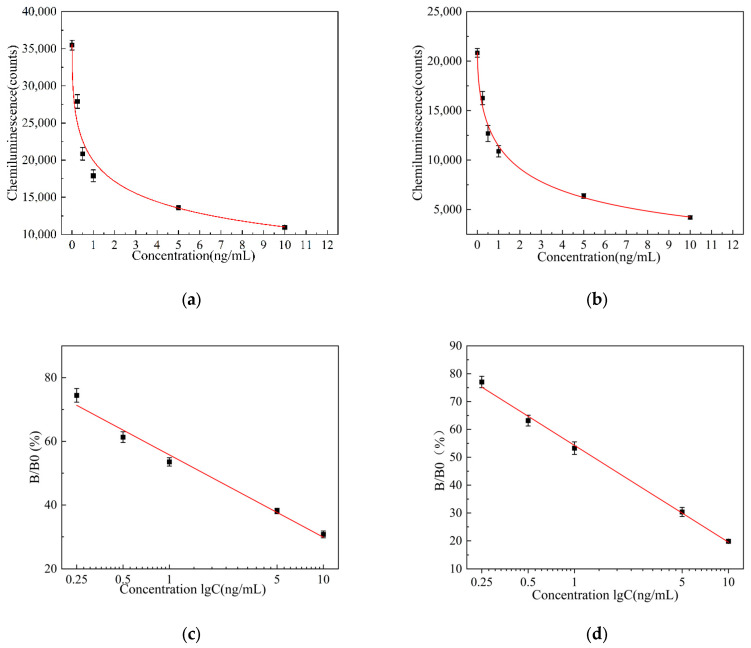
(**a**) Luminescence intensity curve of AFB_1_ standard sample solution in microplate; (**b**) luminescence intensity curve of AFB_1_ standard sample solution in magnetic particles; (**c**) relative luminescence intensity calibration curve of AFB_1_ in microplate; and (**d**) relative luminescence intensity calibration curve of AFB_1_ in magnetic particles.

Under the conditions of the aforementioned pH, aptamer concentration, competitive incubation time, competition incubation temperature, and HRP-labeled antibody concentration, the chemiluminescence intensity was studied for two solid-phase carriers, microplates, and magnetic particles after adding different concentrations of AFB_1_. The average values of the chemiluminescence counts of the AFB_1_ standard sample solutions at three different concentrations after 10 min were calculated, as well as the average values and standard deviations of the three experiments. A plot graph of the luminescence intensity curves of AFB_1_ standard sample solutions at different concentrations, with AFB_1_ concentration as the *x*-axis and chemiluminescence count values as the *y*-axis, was created. The luminescence intensity curves of AFB_1_ standard sample solutions at different concentrations coated on microplates are shown in Figure 5a and those coated on magnetic particles are shown in Figure 5b. The experimental results show that within the range of 0.25–10 ng/mL, as the AFB_1_ concentration increases, different solid-phase carriers exhibit a significant decrease in chemiluminescence intensity with the increasing AFB_1_ concentration. By fitting the data using a four-parameter logistic model, the luminescence curve equation for AFB_1_ standard sample solutions at different concentrations on microplates was obtained as follows:

(1) $$y=-315.07888+35893.29082/(1+(x/{1.77832)}^{0.44836}),R^{2}=0.9877$$

The luminescence curve equation expressions for AFB_1_ standard sample solutions of different concentrations on magnetic microparticles are as follows:

(2) $$y=-1950.49732+22822.91535/(1+(x/{1.82066)}^{0.57982}),R^{2}=0.9974$$

The luminescence curve equations obtained from different concentrations of AFB_1_ standard sample solutions of microtiter plate and magnetic particles show a higher consistency than those obtained from the microtiter plate as a solid-phase carrier, and the magnetic particles as solid-phase carriers show a higher R^2^.

The test results show that the highest chemiluminescence value is obtained when the concentration of the standard sample solution is 0 ng/mL. Using 0 ng/mL as the reference value, the relative luminescence intensity is introduced to plot a relationship curve between the logarithmic value of the AFB_1_ concentration (ng/mL) in the standard sample solution. The relative light intensity expression is as follows:

(3) RLU=B/B0×100%

In the formula, B is the chemiluminescence count value of standard samples of different concentrations of AFB_1_, and B0 is the chemiluminescence count value of the blank standard sample.

The mean values and standard deviations of the relative light intensity (B/B0) were calculated at three different concentrations. A calibration curve of relative light intensity versus the logarithmic values of AFB_1_ concentration was plotted, with the logarithmic values of AFB_1_ concentration as the *x*-axis and relative light intensity as the *y*-axis. The calibration curve of the relative luminescence intensity of AFB_1_ coated on microplates is shown in Figure 5c, and the calibration curve of the relative luminescence intensity of AFB_1_ coated on magnetic particles is shown in Figure 5d. By linearly fitting the data, the calibration curve equation for the relative luminescence intensity of AFB_1_ at different concentrations on the microplate is expressed as follows:

(4) $$y=-25.87552logx+55.76269,R^{2}=0.98338$$

The calibration curve equation for the relative luminescence intensity of AFB_1_ at different concentrations on magnetic microparticles is expressed as follows:

(5) $$y=-34.77077logx+54.27157,R^{2}=0.99679$$

Magnetic particles are characterized by superparamagnetic properties, a high specific surface area, and surface modifiable functional groups. This greatly increases the amount of antigen or antibody binding and improves detection sensitivity. Due to its superparamagnetic properties, it can be more efficiently combined and more thoroughly washed to improve the accuracy of detection. The calibration curve equations for the relative luminescence intensity of AFB_1_ at different concentrations of microtiter plates and magnetic particles show that the curve equations obtained from magnetic particles as solid-phase carrier inclusion aptamers show a higher consistency than those obtained from microtiter plates as solid-phase carrier inclusion aptamers, and the magnetic particles as solid-phase carrier inclusion aptamers show a higher R^2^.

Equations (4) and (5) for the luminescence and calibration curves indicate that the encapsulation of aptamers within magnetic particles, which serve as solid-phase carriers, demonstrate enhanced accuracy, linearity, and overall performance. The performance evaluation curves for both groups are illustrated in Figure 5.

### 3.5. Comparison of Different Chemiluminescent Aptamer Sensors

According to the standard curve equation for the relative luminescence intensity of magnetic microparticles of AFB_1_, the detection limit is calculated using the standard deviation based on the intercept (δ) and the standard curve slope method (S), with the formula as follows:

(6) LOD=3.3δ/S

In Formula 6, δ represents the deviation of the response value. It can be substituted with the standard deviation of the blank group, the residual standard deviation of the standard curve, or the standard deviation of the intercept. The slope (S) of the standard curve was utilized to calculate the limit of detection (LOD) for magnetic particles, which yielded a value of 0.067 ng/mL based on the standard deviation of the intercept. When compared to other types of aptamer sensors, this sensor demonstrated an excellent performance in detecting AFB_1_. Notably, the LOD of this sensor exceeds that of most chemiluminescent sensors used for AFB_1_ detection, resulting in improved detection efficiency. The comparison results with other sensors are shown in Table 1.

### 3.6. Real Sample Analysis

To further validate the applicability of this study in actual samples, this experiment evaluated the spiked recovery rate in corn samples. The standard addition method was used to evaluate the application of this study in corn samples. Totals of 0.5 ug/kg, 1 ug/kg, 2 ug/kg, 5 ug/kg, and 10 ug/kg of AFB_1_ were added to the pure sample solution, respectively. The assay concentration was repeated three times at different spiked concentrations, and the mean values of the assay concentration and recovery at different spiked concentrations, as well as the standard deviation, were obtained. The test results are shown in Table 2, with recovery rates ranging from 94.4% to 108.05%. The results demonstrate that the methods in this study are effective in analyzing the concentration of AFB_1_ in actual samples.

## 4. Conclusions

In this study, we developed an aptamer-antibody chemiluminescent biosensor for the detection of AFB_1_, utilizing an aptamer in place of a traditional antibody. This biosensor is based on the indirect competitive sandwich assay principle and incorporates signal amplification, specifically applied to corn samples. The sensor operates on the principle that AFB_1_ competes with AFB_1_-OVA for binding to the aptamer. The chemiluminescence intensity of the resulting complex—comprising magnetic beads, an aptamer, AFB_1_-OVA, rabbit anti-OVA antibody, and HRP-labeled goat anti-rabbit IgG was detected using a custom-built chemiluminescence detection device. As the concentration of AFB_1_ increases, fewer AFB_1_-OVA complexes are formed, resulting in a decrease in the signals generated by the enzyme reaction products. This methodology can be adapted for the detection of other mycotoxins by simply altering the sequence of the corresponding aptamer and obtaining the specific antibody. Under optimized experimental conditions, we measured the chemiluminescence curves for various concentrations of AFB_1_ and established linear equations correlating the AFB_1_ concentration with chemiluminescence intensity, thus determining the sensor’s lowest detection limit. Compared to other sensor types, this biosensor is characterized by a low cost, high sensitivity, low detection limit, and good recovery rates. The detection linear range spans from 0.25 to 10 ng/mL, with a detection limit of 0.067 ng/mL. The sensor effectively analyzed AFB_1_ in corn samples, yielding recoveries between 94.4% and 108.05%. This sensor serves as a reference for the future development of aptamer-based sensors aimed at the low-cost, high-sensitivity detection of AFB_1_.

## Figures and Tables

**Table 1:** Comparison of this sensor with other types of sensors.

Immunoassay Method	Linear Range (ng/mL)	LOD (ng/mL)	References
Chemiluminescent aptamer	0.25~10	0.067	This work
Chemiluminescent aptamer	0.5~40	0.2	[29]
Chemiluminescent aptamer	0.1~10	0.09	[30]
Chemiluminescent aptamer	1~100	2.85	[31]
Chemiluminescent antibody	0.67~100	0.075	[32]
Fluorescent aptamer	1~100	0.07	[33]
Fluorescent aptamer	10~100	4.1	[34]
Electrochemical aptamers	0.5~200	0.17	[35]

**Table 2:** Determination of the spiked recovery rate of AFB_1_ in samples (n = 3).

Added AFB_1_ (ug/kg)	Detection AFB_1_ (ug/kg)	Recovery (%)
0.5	0.483 ± 0.033	96.6 ± 6.6
1	0.944 ± 0.086	94.4 ± 8.6
2	2.161 ± 0.167	108.05 ± 8.35
5	4.880 ± 0.296	97.6 ± 5.92
10	10.366 ± 0.372	103.66 ± 3.72

## Data Availability

The data presented in this study are available on request from the corresponding authors.
